# Relapse treatment with low-dose steroids in steroid-sensitive minimal change disease

**DOI:** 10.3389/fneph.2024.1426156

**Published:** 2024-07-11

**Authors:** Irene Martin Capon, Eduardo Gutierrez, Ana Huerta, Elizabeth Viera, Marta Alvarez Nadal, Milagros Fernández-Lucas, Javier Villacorta

**Affiliations:** ^1^ Department of Nephrology, Hospital Universitario Ramón y Cajal, Instituto Ramón y Cajal de investigación Sanitaria (IRYCIS), Madrid, Spain; ^2^ Department of Nephrology, Hospital Universitario 12 de Octubre, Madrid, Spain; ^3^ Instituto de Investigación del Hospital Universitario 12 de Octubre (imas12), Madrid, Spain; ^4^ Department of Nephrology, Hospital Universitario Puerta de Hierro, Majadahonda, Madrid, Spain

**Keywords:** minimal change disease, proteinuria, recurrence, remission, steroids

## Abstract

**Background:**

The treatment of minimal change disease (MCD) consists of a high dose of steroids for several months, implying significant drug toxicity. Nevertheless, relapses of steroid-sensitive MCD usually respond to lower doses of steroids.

**Methods:**

The objective of this study was to analyze whether a low dose of steroids (LDS) is effective for the treatment of MCD relapses. Since 2018, new relapses of steroid-sensitive adult patients with MCD in three Spanish centers have been treated with LDS. The cumulative dose of steroids, the time to remission, and the relapse-free time were compared between relapses treated with LDS and previous relapses of the same patients treated with a standard dose of steroids (SDS).

**Results:**

A total of 51 relapses in 31 patients were treated with LDS and compared with 48 historical relapses of the same patients treated with SDS. The mean doses of prednisone adjusted by weight for the initial treatment were 0.45 mg/kg (0.40–0.51 mg/kg) in the relapses treated with LDS and 0.88 mg/kg (0.81–1.00 mg/kg) in those treated with SDS. The mean cumulative doses of prednisone in LDS- and SDS-treated relapses were 1,191 mg (801–1,890 mg) and 3,700 mg (2,755–5,800 mg), respectively. The duration of treatment was 63 days (42–117 days) in the LDS group and was 140 days (65–195 days) in the SDS group. All patients achieved complete remission within 1 month after steroid therapy in both groups. The times to remission of the LDS and SDS groups were 19.10 ± 12.80 and 18.93 ± 12.98 days, respectively (*p* = 0.95).

**Conclusion:**

Among the steroid-sensitive patients with MCD, relapse therapy with LDS (0.5 mg/kg) appears effective and allows minimization of the steroid cumulative dose.

## Introduction

Minimal change disease (MCD) is a common cause of nephrotic syndrome in adults that is characterized by normal glomeruli in light microscopy, diffuse foot process effacement on electron microscopy, and the absence of staining on immunofluorescence (1). The treatment of MCD consists of a high dose of steroids for several weeks, implying significant drug toxicity. However, the dose and the duration of steroid treatment are not standardized, and no controlled studies analyzing different steroid regimens have been performed (2). Clinical guidelines recommend similar therapy for both the initial flare and the subsequent relapses, which consists of high doses of prednisone (1 mg/Kg/day) for 4 weeks or until remission is achieved, with a maximal dose of 80 mg/day. Subsequently, glucocorticoids are tapered by 5–10 mg per week after remission has been achieved for a total period of glucocorticoid exposure of approximately 24 weeks (3).

In recent years, several studies have aimed at minimizing the cumulative dose of corticosteroids for the treatment of MCD. In a prospective multicenter cohort study, Ozeki et al. (4) analyzed therapy with a low dose of steroids (LDS) compared with a standard dose of steroids (SDS) for the treatment of the first MCD relapse. There were no differences in the remission rates between groups, and therapy with LDS was not associated with a greater development of subsequent relapses. In addition, in another observational study developed by the same group, the administration of a shorter course of steroid therapy within 2 months was effective and was not associated with an increased incidence of relapses (5).

The most extensive experience with LDS treatment for MCD was described in the pediatric population. The IPNA (International Pediatric Nephrology Association) recommendation from 2023 suggests that LDS may be started after 4 weeks of prednisone at 60 mg/m^2^ or 2 mg/kg (maximum dose, 60 mg/day), by alternate day prednisone at 40 mg/m^2^ or 1.5 mg/kg (with a maximum dose of 40 mg on alternate days) for 4 weeks (6).

All mentioned studies included a limited number of patients and only evaluated the effect of LDS therapy in an Asian population. The aim of this study was to analyze whether LDS is effective as therapy for relapses in a cohort of adult Caucasian patients with relapsing steroid-sensitive MCD.

## Methods

### Study population and design

**Figure 1:**
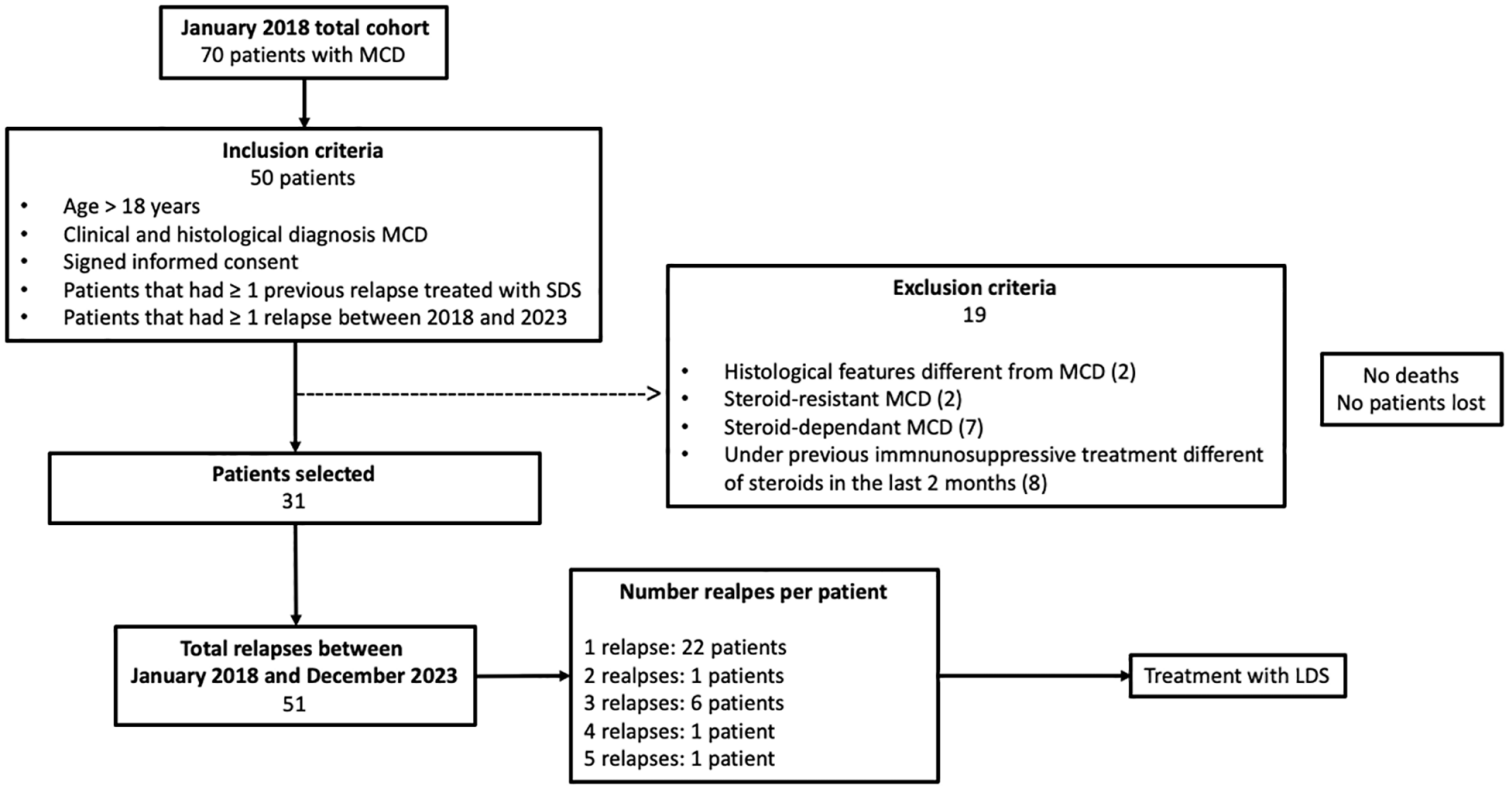
Cohort selection process. Of the 70 adult patients with minimal change disease (MCD) in the cohort in January 2018, we identified 50 patients who had one or more relapses between January 2018 and December 2023. A total of 19 patients were excluded for various reasons. In the end, 31 patients were included in the study. The total number of relapses between January 2018 and December 2023 were 51. There were 11 patients who required additional immunosuppressive treatment. Only one patient that required additional immunosuppression had a new relapse after this treatment, and this relapse was not included in the statistical analysis. *MCD*, minimal change disease; *SDS*, standard-dose steroids; *LDS*, low-dose steroids.

This is a retrospective and multicenter cohort study including three nephrology centers: Hospital Universitario Ramón y Cajal (Madrid), Hospital Universitario 12 de Octubre, (Madrid), and Hospital Universitario Puerta de Hierro (Madrid). In January 2018, there were 70 patients with MCD under follow-up at these centers. Of these patients, 50 met the inclusion criteria for the study: over 18 years old; with a histological confirmation of MCD; who had previously had one or more steroid-sensitive flares of the disease; and who had at least one disease relapse between January 2018 and December 2023 (**Figure 1**). A total of 19 patients were excluded for various reasons: other histological features different from MCD (*n* = 2); with steroid-resistant MCD (*n* = 2); with steroid-dependent MCD (*n* = 7); and those who required previous immunosuppressant therapy other than steroids (*n* = 8). In the end, 31 patients were included in the study. Of these, 22 had one relapse, one patient had two relapses, six patients had three relapses, one patient had four relapses, and one patient experienced five relapses. All of the relapses analyzed occurred after complete remission of a previous flare and without receiving any immunosuppressant treatment within the previous 2 months. During the follow-up, when the patients experienced a MCD relapse, this was treated with a LDS regimen (0.5 mg/Kg/day of prednisone) for 4 weeks or until remission was achieved, followed by a rapid tapering of the steroid dose within 3 months (tapering 10 mg every week up to 5 mg, then 5 mg/day for 1 week and withdrawn). The initial prednisone dose was adjusted according to the patient’s dry weight, i.e., the weight immediately prior to the MCD flare.

**Figure 2:**
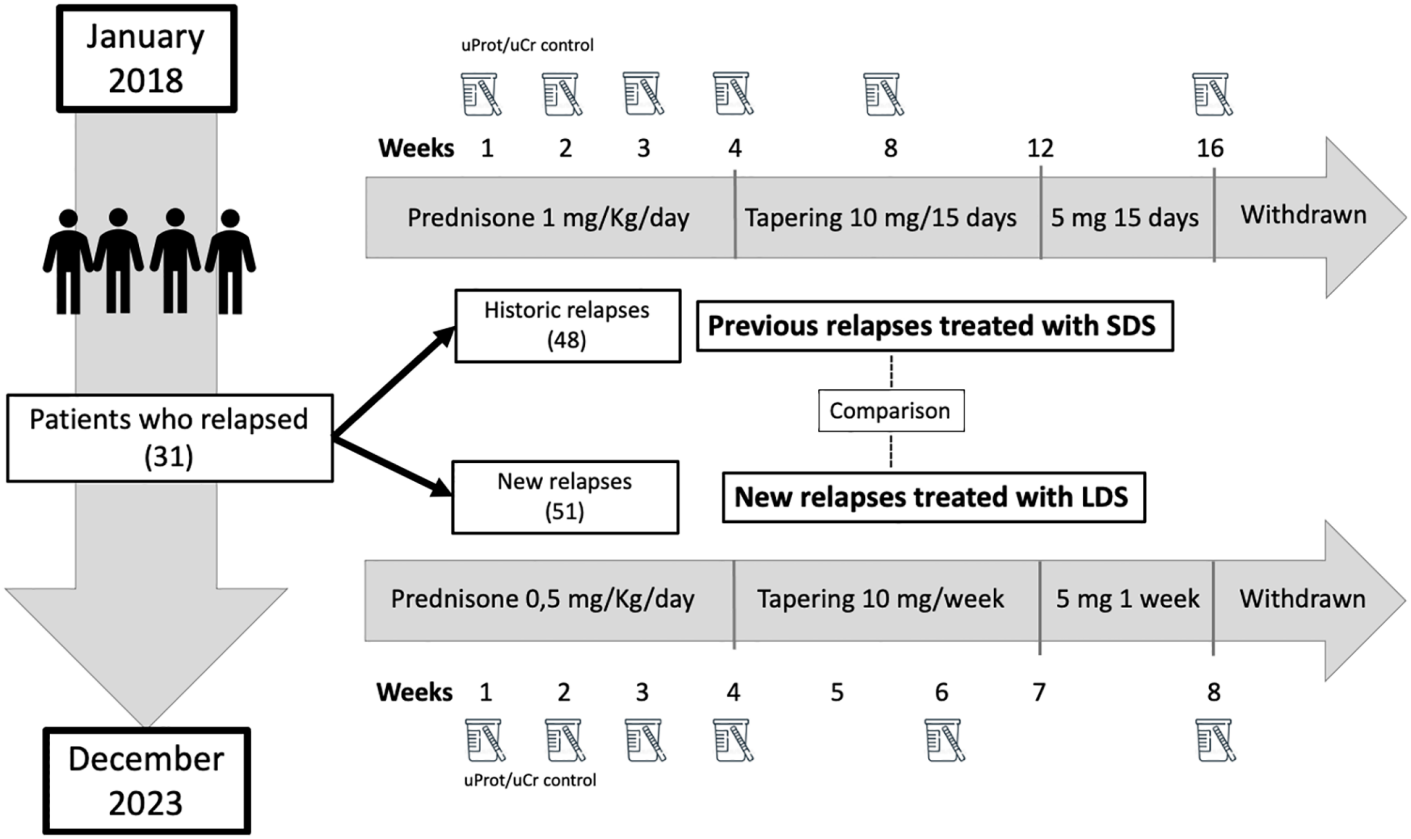
Treatment protocol for minimal change disease relapses. *LDS*, low-dose steroids; *SDS*, standard-dose steroids; *uProt/uCr*, urine protein/creatinine excretion ratio.

MCD flares treated with a LDS regimen were compared with the previous flares of the same patients treated with standard therapy consisting of 1 mg/Kg/day of prednisone for at least 4 weeks and then tapering 10 mg each 15 days to 5 mg, then 5 mg/day for 1 week and withdrawn (**Figure 2**).

This study was performed in accordance with the Declaration of Helsinki. All participants provided written informed consent for the study, which was approved by the Ethics Committee from Hospital Ramon y Cajal.

### Clinical and laboratory data

Baseline data at the onset of each relapse and during follow-up were compiled from the medical records of all participating centers, following a uniform protocol that included demographics, clinical presentation, therapeutic management, and laboratory parameters that were deemed of interest [i.e., age, sex, weight (in kilograms), urine protein/creatinine excretion ratio (uProt/uCr) (in milligrams per gram), serum creatinine (sCr) (in milligrams per deciliter), estimated glomerular filtration rate (eGFR) measured using the Chronic Kidney Disease Epidemiology Collaboration (CKD-EPI) equation (in milliliters per minute per 1.73 m^2^) (7), serum albumin (in grams per liter), prednisone dose adjusted to weight (in milligrams per kilogram), type of immunosuppression employed before and after the relapse, time to remission (in days), cumulative dose of prednisone at the end of the flare (in milligrams), and time to further relapse (in months)].

### Outcomes and definitions

The primary outcome was the percentage of patients who achieved remission on day +30 after therapy. Secondary outcomes included time to achieve remission, the cumulative steroid dose employed in each flare, relapse-free survival after therapy, and steroid side effects.

MCD remission was defined as a reduction of proteinuria <300 mg/day, uProt/uCr <300 mg/g, and/or negative result for urinary protein on a dipstick test. Steroid-sensitive patients were those patients with MCD who presented complete remission after 4 weeks of prednisone. MCD relapse was defined as the recurrence of protein >500 mg/day or uProt/uCr >500 mg/g. When patients detected relapse symptoms (e.g., edema, foamy urine, or weight gain), a quantitative urine measurement was immediately requested to define the degree of proteinuria. Frequently relapsing patients were those patients with two or more relapses within 6 months or four or more relapses within 12 months. Relapse-free survival was considered as the relapse-free time since the patient achieved complete remission in the previous flare. The cumulative dose of steroid was calculated as the sum of the daily dose of prednisone from the start of the treatment to the end of the tapering.

Data on steroid side effects were collected retrospectively. When an adverse effect occurred, it was recorded in the corresponding flare, whether treated with SDS or LDS. The definitions of steroid side effects were as follows: steroidal diabetes was defined as an increase in blood glucose associated with glucocorticoid treatment, with random plasma glucose of ≥200 mg/dL or HbA_1c_ ≥ 6.5% with symptoms of hyperglycemia. Mild infection was defined as an infection that did not require hospital admission, while severe infection was defined as an infection that did require hospital admission. The psychiatric side effects of steroid use included confusion, agitation, perplexity, hallucinations, and delusions or cognitive impairment, occurring within the first 10 days after the initiation of treatment.

### Statistical analysis

Categorical data were expressed as absolute numbers and percentages, while continuous data were expressed as the mean and standard deviation (SD). In the case of non-normal distribution, data were expressed as the median and interquartile range (IQR). The Shapiro–Wilk test was used to test for normality. Comparisons were made with the *t*-test for normally distributed continuous variables and with the Mann–Whitney *U* test for the non-normally distributed continuous variables. The chi-square or Fisher’s exact test was employed to compare the qualitative variables. The statistical significance level was set at *p* < 0.05. As the same patient can have several flares and each flare can have a different treatment, a repeated-measures regression model (GEE model) with interchangeable structure was applied to compare the flares. Kaplan–Meier survival analysis and the long-rank test were used to compare relapse-free survival and the cumulative incidence of relapse after remission in the LDS- and SDS-treated flares, respectively. Statistical analyses were conducted using IBM SPSS version 22.0.

## Results

### Treatment

All new relapses (*n* = 51) were treated with LDS and were compared with 48 previous relapses of the same patients treated with SDS. The main clinical and laboratory features of the population at the onset of each flare are detailed in **Tables 1**, **2**.

**Table 1:** Clinical characteristics at baseline of the study population.

Variable	*n* = 31
**Sex, *n* (%)**	12 (40) women 19 (60) men
**Age (years), *M* ± SD**	43.1 ± 19.7
**Body weight at the onset of flare (kg), *M* ± SD**	74.27 ± 15.04
**BMI (kg/m^2^), *M* ± SD**	24.2 ± 5.1
**HTN, *n* (%)**	2 (10)
**DM, *n* (%)**	1 (5)
**Chronic kidney disease, *n* (%)**	0 (0)
**Relapses treated, *n***	SDS = 48 LDS = 51
**Median follow-up since disease onset (months), (IQR)**	113.8 (30.2–135.7)
**Median follow-up since LDS treatment (months), (IQR)**	50.9 (10.5–57)

Continuous data are expressed as the mean (M), standard deviation (SD), and interquartile range (IQR). Categorical variables are expressed as n (%).

BMI, body mass index; DM, diabetes mellitus; HTN, hypertension; SDS, standard-dose steroids; LDS, low-dose steroids.

**Table 2:** Biochemical features at the onset of minimal change disease (MCD) flares treated with low-dose steroids (LDS) and standard-dose steroids (SDS).

Variable	LDS	SDS	*p*
**Median urinary protein/creatinine ratio (mg/g), *M* ± SD**	6,559.19 ± 5,182.01	6,905.78 ± 3,084.45	0.86
**Mean serum albumin (mg/dL), *M* ± SD**	2.55 ± 0.86	2.07 ± 0.69	0.06
**Mean baseline SCr (mg/dL), *M* ± SD**	0.94 ± 0.42	0.91 ± 0.29	0.67
**Mean baseline eGFR (mL/min per 1.73 m^2^), M ± SD**	93.79 ± 23.40	96.81 ± 21.36	0.55
**Mean cholesterol (mg/dL), *M* ± SD**	263.34 ± 84.74	234.97 ± 102.17	0.08

Continuous data are expressed as the mean (M) and standard deviation (SD).

SCr, serum creatinine; eGFR, estimated glomerular filtration rate [Chronic Kidney Disease Epidemiology Collaboration (CKD-EPI) equation].

The median initial doses of prednisone for relapse therapy were 35 mg/day (30–40 mg/day) in patients treated with LDS and 60 mg/day (60–77 mg/day) in those treated with SDS [GEE model: −45.6 (−58.8 to −31.72)]. The median doses of prednisone adjusted by body weight in LDS- and SDS-treated relapses were 0.45 mg/Kg/day (0.40–0.53 mg/Kg/day) and 0.88 mg/Kg/day (0.81–1 mg/Kg/day), respectively (*p* < 0.05). The median cumulative doses of prednisone in patients treated with LDS and SDS were 1,191 mg (801–1,890 mg) and 3,700 mg (2,755–5,800 mg), respectively (*p* < 0.05). The median durations of therapy in the LDS and SDS groups were 63 days (42–117 days) and 140 days (65–195 days), respectively [GEE model: −54 days (−106 to −3.45)].

### Outcome measurement

**Figure 3:**
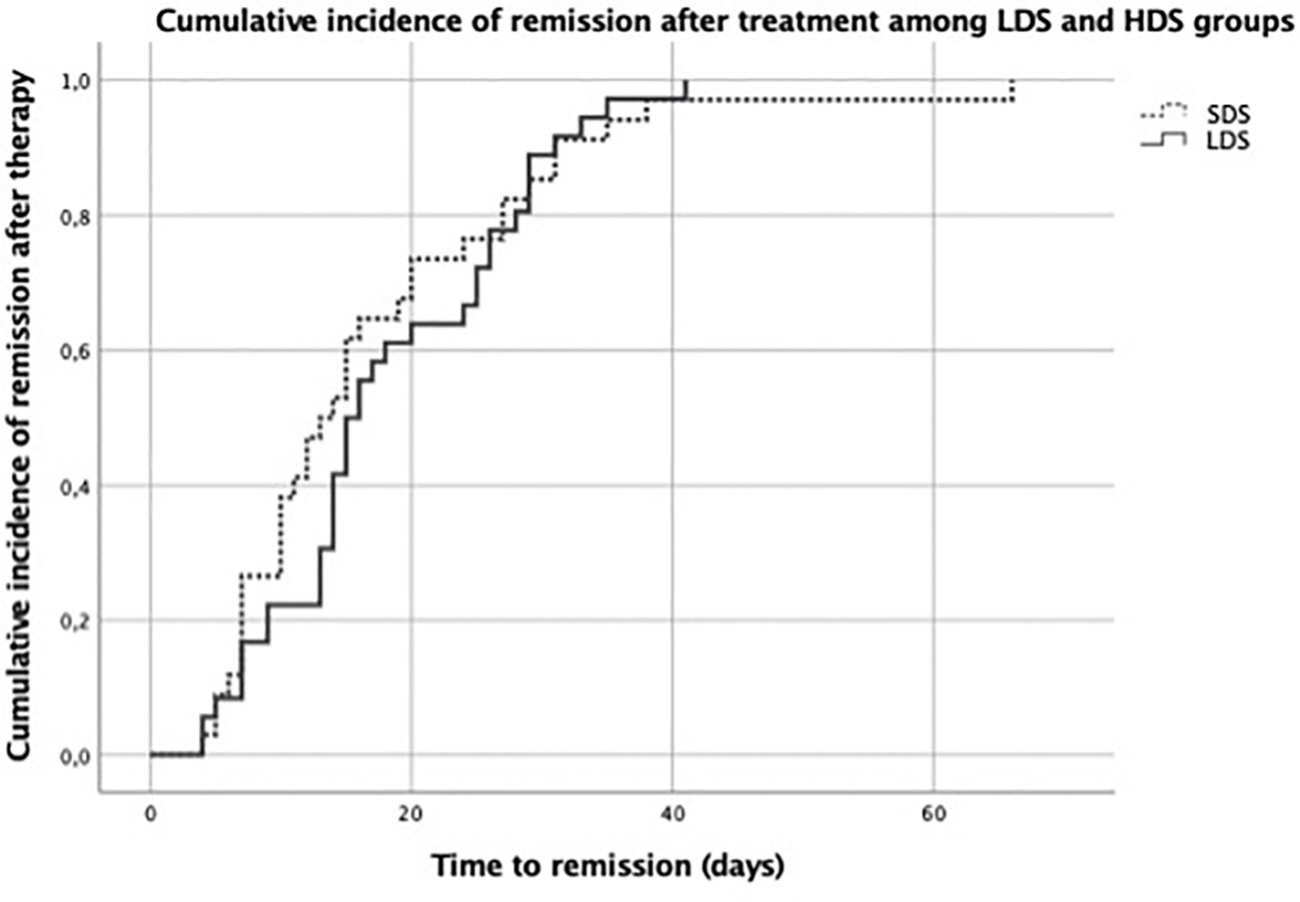
Cumulative incidence of remission after treatment in the low-dose steroid (LDS) and standard-dose steroid (SDS) groups.

All patients (100%) achieved complete remission after 1 month of steroid therapy in both LDS- and SDS-treated relapses (**Table 3**). The cumulative incidence of remission after treatment in LDS- and SDS-treated relapses is shown in **Figure 3**. The mean time to remission was 19.10 ± 12.80 days in LDS-treated-relapses and was 18.93 ± 12.98 days in SDS-treated relapses [GEE model: −0.5 (−5.18 to 4.18)]. Data on the appearance of side effects associated with steroid treatment in each treatment group are reflected in **Table 4**.

**Table 3:** Outcomes of patients with minimal change disease (MCD) treated with low-dose steroids (LDS) and standard-dose steroids (SDS).

Variable	LDS	SDS	*p*
**Percentage of remission, *n* (%)**	48 (100)	51 (100)	–
**Mean time to remission (days), *M* ± SD**	19.10 ± 12.80	18.93 ± 12.98	0.95

Continuous data are expressed as the mean (M) and standard deviation (SD).

**Table 4:** Steroid side effects after treatment.

			Total no. of patients (*N* = 31)	Total relapses (*N* = 99)	LDS-treated relapses (*N* = 51)	SDS-treated relapses (*N* = 48)
Steroid side effects	No		21 (67%)	89 (90%)	45 (88%)	44 (91%%)
	Yes		10 (33%)	10 (10%)	6 (12%)	4 (8%)
		Steroidal diabetes	3 (10%)	3 (3%)	2 (4%)	1 (2%)
		Mild infection	5 (16%)	5 (5%)	3 (6%)	2 (4%)
		Severe infection	0	0	0	0
		Psychiatric effects	2 (6%)	2 (2%)	1 (2%)	1 (2%)

Categorical variables are expressed as n (%).

LDS, low-dose steroids; SDS, standard-dose steroids.

**Figure 4:**
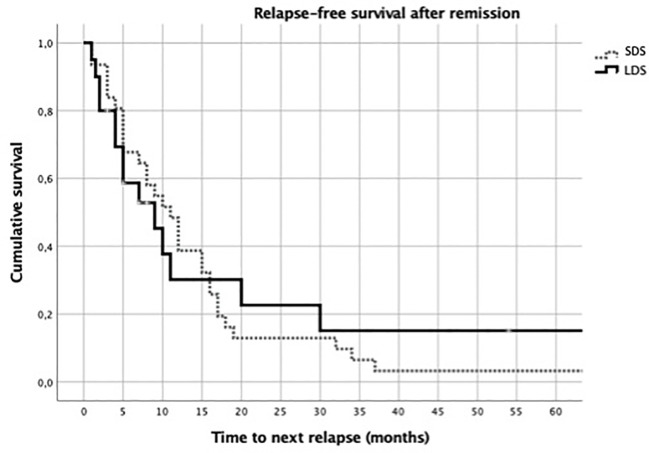
Relapse-free survival after remission in the low-dose steroid (LDS) and standard-dose steroid (SDS) groups.

Among patients who relapsed more than once, the median relapse-free survival was 5 months (3.25–9.75 months) in the LDS group and was 8 months (4–15 months) in the SDS group (*p* = 0.36). The relapse-free survival after remission was comparable between the LDS- and SDS-treated relapses (**Figure 4**).

During the follow-up, 11 of the 31 patients required another additional immunosuppressive drug due to the development of a steroid-dependent MCD flare (*n* = 9) or the development of frequently relapsing MCD (*n* = 2). The type and dosage of immunosuppression were chosen according to the clinician criteria. Six patients received mycophenolate mofetil, four patients were given rituximab, and one patient was administered cyclophosphamide. Further relapses after the second line of immunosuppressive therapy were not included in the study. In patients who experienced more than three relapses since the start of the study, but did not meet the criteria for frequent relapsers, the initiation of another steroid-sparing immunosuppressive drug was considered. However, this was dismissed either due to patient refusal or high risk of infection. There were no deaths and no patients lost during the follow-up.

## Discussion

Steroids are the cornerstone of the treatment for MCD, and the response to steroid therapy at the onset of the disease determines the prognosis of the glomerular disease. Current guidelines recommend treatment with high-dose prednisone during several weeks for both the initial flare and the subsequent relapses of MCD in adults. In the case of the initial flare, recommendations are based on retrospective series and the evidence obtained from pediatric clinical trials, whereas the recommendations for relapse therapy are extrapolated from the results of mentioned studies (8–10). In a non-randomized pilot clinical trial, Zion et al. demonstrated the feasibility of a shortened- and lowered-dose steroid regimen in a pediatric cohort. Patients in the intervention group did not have a higher number of relapses or more steroid side effects (11). Notably, the only clinical trial performed in adults is more than 50 years old and compared LDS therapy against no specific treatment for the initial flare of MCD (12).

On the other hand, the widely known side effects of steroids have led to the exploration of new strategies that minimize the dose of corticosteroids utilized for MCD treatment (13). To date, the most widely employed strategy to minimize the steroid dose at the initial flare is based on the combination of LDS with non-steroidal immunosuppressants such as mycophenolate mofetil (14, 15), tacrolimus (16), cyclophosphamide (17), cyclosporin (18), and the recently proposed rituximab (19, 20). Despite the recurrent disease profile of this podocythopathy, only a limited number of studies have evaluated steroid minimization strategies for relapse therapy.

In the present study, we demonstrated the effectiveness of the LDS regimen without any additional immunosuppressants for MCD relapse. All relapses treated with LDS in our series achieved complete remission, and the time to remission was comparable to that of relapses that received SDS therapy. These results suggest that MCD relapses could be treated effectively with LDS without the addition of another immunosuppressive treatment and any delay in renal response. Consequently, a significant lower cumulative exposure to steroids was observed in patients treated with LDS. In our series, the mean cumulative dose of prednisone used per flare in these patients was almost one-third of that employed in patients treated with the standard dose of prednisone. Therefore, this significant reduction in prednisone exposure might imply fewer adverse effects and less drug toxicity. In this study, the steroid-derived side effects did not differ among patients treated with LDS and SDS, and no severe infections were recorded. On the other hand, up to 10% of patients developed diabetes mellitus during follow-up, and the causative association between steroid exposure and diabetes is well established. Moreover, although there were no significant differences in the side effects of steroids in both groups, it should be considered that this study did not compare patients treated with SDS and different patients treated with LDS. As the same patients were treated with different doses, in the low-dose-treated flares, there has already been a previous high cumulative steroid dose, which could have conditioned the development of more side effects in the LDS-treated flares.

We believe that LDS monotherapy represents a fundamental shift in the treatment of relapses in MCD. While additional immunosuppressive strategies have been developed in recent years to reduce the risk of disease recurrence, many patients either experience relapse despite already being on immunosuppressive therapy or are unwilling to add another treatment to their regimen. Thus, monotherapy with LDS may be effective when aiming to avoid adding another immunosuppressive treatment.

On the other hand, in this study, there appears to be a non-statistically significant trend in patients treated with LDS where the time to the next flare is shorter than that when treated with SDS. Nevertheless, it should be kept in mind that this does not imply a higher risk of relapse, as up to 64% of patients do not relapse after treatment with LDS.

This study has some limitations, such as the small sample size and the differences in the serum albumin between relapses treated with LDS and SDS. Although this difference was not statistically significant and proteinuria was similar in both groups, patients treated with SDS might have exhibited greater severity of the nephrotic syndrome. We believe that an earlier diagnosis and an early referral to a nephrologist for relapses treated with LDS could explain these differences as the LDS regimen is only employed in flares after a longer follow-up, when patients are more aware of possible recurrence and trained for early diagnosis. Therefore, it cannot be excluded that this fact could have influenced the response rates to LDS. However, to the best of our knowledge, this is one of the few published studies analyzing the different steroid regimens for relapse therapy in adult Caucasian patients with MCD and reporting the efficacy of the LDS regimen in this population.

In conclusion, among steroid-sensitive adult MCD patients, relapse therapy with LDS (0.5 mg/kg) appears effective and allows significant minimization of the steroid cumulative dose. Further controlled studies are necessary to test the efficacy and safety of different steroid doses in adult MCD.

## Data availability statement

The raw data supporting the conclusions of this article will be made available by the authors, without undue reservation.

## Ethics statement

The studies involving humans were approved by Department of Nephrology, University Hospital Ramon y Cajal. The studies were conducted in accordance with the local legislation and institutional requirements. The participants provided their written informed consent to participate in this study.

## Author contributions

IM: Conceptualization, Data curation, Formal analysis, Investigation, Methodology, Software, Writing – original draft, Writing – review & editing. EG: Conceptualization, Data curation, Investigation, Methodology, Supervision, Validation, Writing – review & editing. AH: Conceptualization, Data curation, Investigation, Supervision, Validation, Writing – review & editing. EV: Conceptualization, Data curation, Formal analysis, Investigation, Methodology, Writing – review & editing. MAN: Conceptualization, Data curation, Formal analysis, Investigation, Methodology, Writing – review & editing. MF-L: Conceptualization, Supervision, Validation, Writing – review & editing. JV: Conceptualization, Data curation, Formal analysis, Investigation, Methodology, Project administration, Supervision, Validation, Visualization, Writing – review & editing, Writing – original draft.
